# Advances in the Regulatory Mechanism of Enzymes Involved in Soluble Sugar Metabolism in Fruits

**DOI:** 10.3390/plants15010138

**Published:** 2026-01-03

**Authors:** Zixin Meng, Weiming Li, Guodi Huang, Xiang Li, Riwang Li, Yongsen Chen, Shixing Luo, Limei Guo, Yingying Tang, Yujuan Tang, Yu Zhang, Xiaowei Ma, Li Li

**Affiliations:** 1 Guangxi Engineering Research Center of Green and Efficient Development for Mango Industry, Guangxi Subtropical Crops Research Institute, Guangxi Academy of Agricultural Sciences, Nanning 530001, China; 2 College of Agriculture, Guangxi University, Nanning 530004, China; 3 Guangxi Key Laboratory for Polysaccharide Materials and Modification, School of Marine Sciences and Biotechnology, Guangxi Minzu University, Nanning 530008, China; 4 Key Laboratory of Tropical Fruit Biology of Ministry of Agriculture, South Subtropical Crops Research Institute, Chinese Academy of Tropical Agricultural Sciences, Zhanjiang 524019, China

**Keywords:** fruit soluble sugars, sugar-metabolizing enzymes, epigenetic regulation, transcriptional regulation, signaling pathways

## Abstract

Soluble sugars are key determinants of fruit quality, directly influencing sensory attributes such as sweetness and flavor, as well as nutritional value and texture. Their content and composition are precisely regulated by sugar-metabolizing enzymes. Key enzymes, including invertase (INV), sucrose phosphate synthase (SPS), sucrose synthase (SUS), fructokinase (FRK), and hexokinase (HXK), play pivotal roles in these processes. However, a systematic and in-depth analysis of their regulatory mechanisms is currently lacking, which hinders a comprehensive understanding of the regulatory network governing fruit sugar metabolism. This review employs bibliometric analysis to systematically examine research trends in fruit sugar metabolism. Furthermore, it synthesizes recent advances in the coordinated regulatory mechanisms from the perspectives of transcriptional regulation, epigenetic modifications, and signal transduction, aiming to provide a clearer framework for future research. At the transcriptional level, transcription factor families such as MYB, WRKY, NAC, and MADS-box achieve precise regulation of sugar metabolism-related genes by specifically binding to the promoters of their target genes. Regarding epigenetic regulation, mechanisms including histone modifications, non-coding RNAs, and DNA methylation influence the expression of sugar-metabolizing enzymes at the post-transcriptional level by modulating chromatin accessibility or mRNA stability. Signaling pathways integrate hormonal signals (e.g., ABA, ethylene), environmental signals (e.g., temperature, light), and sugar-derived signals into the regulatory network, forming complex feedback mechanisms. These regulatory mechanisms not only directly affect sugar accumulation in fruits but also participate in fruit quality formation by modulating processes such as cell turgor pressure and carbon allocation. By integrating recent findings on transcriptional regulation, epigenetics, and signaling pathways, this review provides a theoretical foundation for fruit quality improvement and targeted breeding.

## 1. Introduction

With the steady growth of the global fruit industry, the focus for most fruits has shifted from yield to quality, making fruit quality a key factor affecting market value and competitiveness [1,2,3]. Among the various factors influencing fruit quality, the content and composition of soluble sugars strongly determine fruit taste and sweetness. The primary soluble sugars in fruits are glucose, fructose, and sucrose, which contribute differently to overall sweetness—fructose is the sweetest, while glucose has the most pleasant flavor [4,5,6]. Sugar metabolism plays a central role in the distribution of photosynthetic products and energy during fruit development. It supplies carbon and energy for fruit growth and dynamically regulates sugar composition and levels through the activities of various enzymes and transport proteins. This process directly affects fruit flavor, storage performance, and commercial value, underscoring its crucial role in fruit quality [7].

Therefore, understanding the regulation of sugar metabolism is of importance for improving fruit quality and guiding the breeding of superior new varieties. This article reviews the roles of key sugar metabolism enzymes and their regulation at multiple levels, including transcriptional control, epigenetic mechanisms, and signaling pathways. The aim is to provide new and useful insights for both research and practical applications in this field.

## 2. Bibliometric Analysis of Research Trends in Fruit Sugar Metabolism

Bibliometric analysis, which provides a data-driven foundation for systematically reviewing research trends, serves as an important tool for understanding developments in scientific disciplines [8,9,10,11]. In line with this approach, the present study employed VOSviewer (1.6.20) to analyze literature on sugar metabolism from the Web of Science database. This analysis aimed to identify research hotspots and trace global publication trends over the past decade. This study utilized the Web of Science Core Collection database and VOSviewer (1.6.20) software to perform keyword co-occurrence analysis of publications related to fruit sugar metabolism from 2016 to 2025. The search was conducted using the title query: (fruit OR apple OR tomato OR grape OR strawberry OR citrus OR peach) AND (sugar OR “soluble sugar” OR sucrose OR fructose OR glucose OR “sugar accumulation” OR “carbohydrate metabolism”). The document type was limited to research articles, and the subject areas were confined to Plant Sciences and Horticulture. The analysis included 1602 publications that met these criteria.

In the keyword processing phase, a minimum occurrence threshold of 30 was established. Synonyms were consolidated while maintaining the most representative terminology. The results are presented through two visualization approaches. A network visualization displays the research structure, while an overlay visualization demonstrates the research evolution throughout the decade.

**Figure 1:**
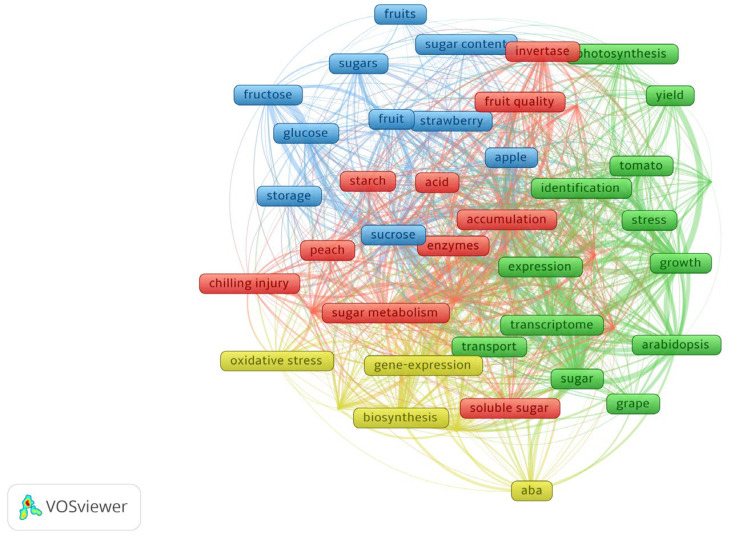
Keyword co-occurrence network analysis of fruit sugar metabolism research. Note: Each frame represents a keyword. The color of the frames represents different thematic clusters.

**Figure 2:**
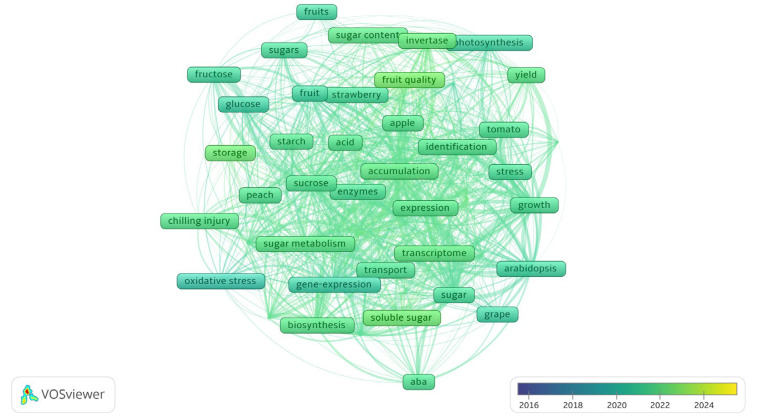
Co-occurrence map of journal keywords (2016–2025). Note: Each frame represents a keyword. The color of a frame indicates the publication year of the documents in which that keyword appeared.

Figure 1 clearly reveals several major research domains. These encompass fundamental investigations into photosynthesis and key metabolic enzymes, studies examining the biosynthesis, transport, and storage of soluble sugars in prominent fruit species including apple, grape, and tomato, as well as research exploring the interactions between sugar metabolism and abiotic stress conditions such as chilling injury, along with hormonal signals like ABA. The visualization reveals “gene expression” and “transcriptome” as the network’s core, linked to all domains. This identifies transcriptional regulation as a key node, central to fruit metabolism, stress resistance, and quality formation. Figure 2 shows from a temporal dimension that recent research has also concentrated on areas such as metabolic enzyme function, soluble sugar accumulation mechanisms, fruit quality, and postharvest storage technology.

Based on these research hotspots, this paper will first explore the core metabolic pathways and their key enzymes, and then systematically elucidate the latest research progress on their regulatory mechanisms from the perspectives of transcriptional regulation, epigenetic modifications, and signal transduction pathways.

## 3. Fruit Sugar Metabolism Pathways and the Function of Key Enzymes Involved

**Figure 3:**
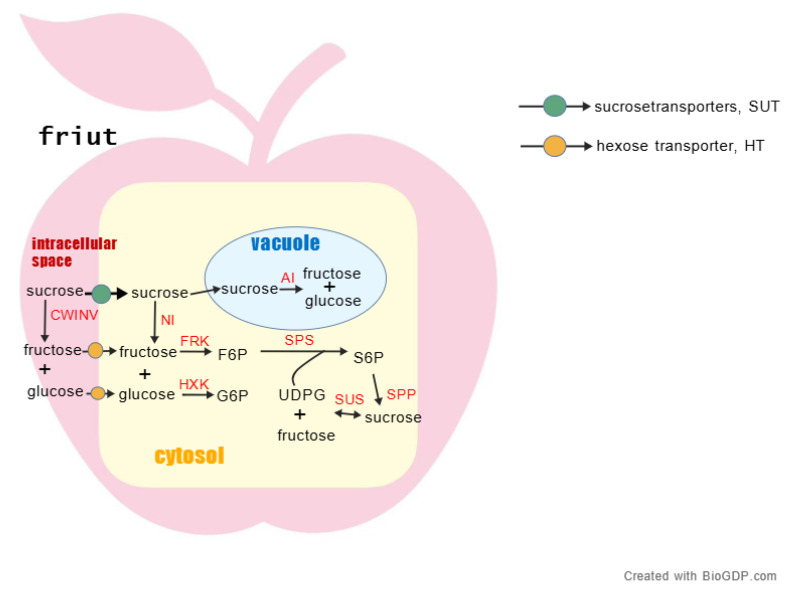
Schematic diagram of key pathways in fruit soluble sugar metabolism. Note: This figure summarizes the interconversion, transmembrane transport, and compartmentalization of major soluble sugars within a fruit cell. Key enzymes are highlighted in red. Sucrose enters the cell from the extracellular space via sucrose transporters (SUTs). It can then undergo irreversible hydrolysis catalyzed by cell wall invertase (CWINV) or neutral invertase (NI), yielding glucose and fructose. Additionally, sucrose can be reversibly cleaved in the cytosol by sucrose synthase (SUS) into fructose and uridine diphosphate glucose (UDPG). Sucrose and glucose enter the cell via hexose transporters (HT). In the cytosol, fructose and glucose are phosphorylated by fructokinase (FRK) and hexokinase (HXK), respectively, to form fructose-6-phosphate (F6P) and glucose-6-phosphate (G6P). Sucrose biosynthesis primarily occurs through sequential catalysis by sucrose phosphate synthase (SPS) and sucrose phosphatase (SPP), utilizing F6P and UDPG as substrates. In the vacuole, sucrose can be stored or hydrolyzed into fructose and glucose by vacuolar acid invertase (AI). Solid arrows in the figure indicate the direction of major metabolic reactions. Abbreviations: AI, acid invertase; CWINV, cell wall invertase; FRK, fructokinase; F6P, fructose-6-phosphate; G6P, glucose-6-phosphate; HXK, hexokinase; NI, neutral/alkaline invertase; SPS, sucrose phosphate synthase; SPP, sucrose phosphatase; SUS, sucrose synthase; SUT, sucrose transporter; UDPG, uridine diphosphate glucose.

Fruits accumulate various soluble sugars, the metabolism of which is primarily categorized into three pathways: sucrose metabolism, hexose metabolism, and sorbitol metabolism. Sucrose metabolism encompasses the synthesis, degradation, and interconversion of sucrose. Hexose metabolism refers to the conversion of photosynthetic products into fructose and glucose. Sorbitol metabolism is predominant in Rosaceae fruits. Key enzymes governing sucrose metabolism include Invertase (INV), Sucrose Phosphate Synthase (SPS), and Sucrose Synthase (SUS), while major enzymes in hexose metabolism comprise Hexokinase (HXK) and Fructokinase (FRK) [12]. Figure 3 illustrates the core pathways of sucrose and hexose metabolism. The following sections will provide a detailed introduction to the key enzymes and their functions in these metabolic pathways.

### 3.1. Key Enzymes in Sucrose Metabolism Pathway

#### 3.1.1. Invertase (INV)

Sucrose unloaded from the phloem into fruits is irreversibly hydrolyzed into glucose and fructose by invertase (INV). This process provides energy for fruit growth and metabolism, directly influencing sweetness and sugar accumulation. Based on their optimal pH, INV are categorized into two main types: acid invertase (AI) and neutral/alkaline invertase (NI). AI is further subdivided by subcellular localization into cell wall invertase (CWINV) and vacuolar acid invertase (VINV) [13,14,15,16].

Different INV isoforms play distinct regulatory roles across fruit developmental stages. During early mango fruit developmental, AI activity maintains high, hydrolyzing sucrose into glucose and fructose to meet the high sugar demand for rapid fruit growth [17]. In strawberry, Yuan et al. found that *FaCWINV1* is specifically highly expressed during the fruit coloring stage, and its mutant exhibits reduced fruit size and sugar content [18]. Topcu et al. observed that INV activity was lowest in green strawberries and relatively higher in pink and red stages, showing a positive correlation with sugar content [19]. In apple, the neutral invertase *MdNINV6*, localized in the cytoplasm, is directly activated by the transcription factor *MdNAC5*, promoting sucrose conversion to fructose [20]. Overexpression of *MdNAC5* increased fruit fructose content by 35%, underscoring the critical role of the NINV subfamily in hexose accumulation. Luo et al. found that in two melon cultivars (‘Fengmi 2’ and ‘Good Luck 52’), fruit development initiated within 16 days after pollination, marked by a clear increase in reducing sugars [21]. Currently, AI and NI activities dropped sharply in both fruits and leaves, followed by rapid sucrose accumulation, indicating a developmental switch to sucrose storage. After 16 days, AI and NI activities continued to decline in the pulp, whereas NI activity increased in the leaves.

Furthermore, INV participated in abiotic stress responses. Under cold and drought conditions, INV helps modulate sugar levels to cope with adverse conditions. During cold storage of peach fruit, the expression of the vacuolar invertase gene *PpVIN2* was significantly induced. The invertase inhibitor *PpINH1* was found to interact with *PpVIN2*, limiting INV activity, elevating cellular sugar levels, and thereby enhancing chilling tolerance [22].

#### 3.1.2. Sucrose Phosphate Synthase (SPS)

SPS is a key rate-limiting enzyme in the sucrose biosynthetic pathway, critically regulating the rate and accumulation of sucrose synthesis. It catalyzes the reaction between fructose-6-phosphate (F6P) and uridine diphosphate glucose (UDPG) to form sucrose-6-phosphate (S6P), which is subsequently dephosphorylated by sucrose phosphatase (SPP) to yield sucrose [23,24].

SPS activity directly influences sucrose accumulation levels in fruits. A study by Gao et al. demonstrated that exogenous ethylene treatment in melon ‘HS’ fruit significantly up-regulated SPS activity and *CmSPS1* gene expression, accompanied by increased sucrose content [25]. In contrast, treatment with the ethylene inhibitor 1-MCP produced the opposite effect, confirming that ethylene signaling influences sucrose accumulation by regulating SPS activity and reinforcing the promotive role of SPS in sucrose biosynthesis. Wang et al. showed that high sucrose accumulation in the late stage of pumpkin ‘312-1’ fruit was closely associated with elevated SPS gene expression, whereas low SPS expression in ‘98-2’ resulted in significantly lower sucrose content [26].

SPS gene expression varies markedly among plant tissues, differentially influencing growth and development across organs. Yan et al. identified three SPS genes—*RuSPS1*, *RuSPS2*, and *RuSPS3*—in blackberry, which were ubiquitously expressed yet displayed distinct spatial expression patterns [27]. Specifically, *RuSPS1* expression was elevated in leaves and fruits and *RuSPS2* was strongly upregulated in fully developed fruits, while *RuSPS3* maintained consistently high expression levels across all organs.

#### 3.1.3. Sucrose Synthase (SUS)

Sucrose synthase (SUS) is the only glycosyltransferase in sucrose metabolism with bidirectional functionality. It reversibly catalyzes the conversion of sucrose into fructose and UDP-glucose, operating in both sucrose cleavage and synthesis. Its activity exhibits tissue-specific functional bias, making SUS a central hub in the sucrose metabolic network [23,28,29].

The bidirectional function of SUS in fruit sugar metabolism varies among cultivars. Zhang et al. identified seven SUS family genes in jujube, among which *Zj029* and *Zj050* were highly expressed [30]. Their study revealed a significant negative correlation between SUS cleavage activity and sucrose content (by *Zj218*), whereas synthetic activity (by *Zj050*) enhanced sucrose accumulation, indicating that SUS maintains sucrose homeostasis through bidirectional regulation. Huang et al. reported that the enzyme activity of sucrose synthase gene *VvSS3* peaked before the véraison stage in grape [31]. During this stage, it primarily functioned in the cleavage direction, directly increasing glucose and fructose levels and enhancing fruit sweetness. Mutations at Ser176 and Ser381 sites of VvSS3 significantly reduced its synthetic activity—by 36.6% and 45.9%, respectively—while enhancing cleavage activity by 66.3% and 12%. These results demonstrate that cleavage is the dominant pathway for sugar regulation by SUS.

Under stress conditions, SUS helps maintain energy homeostasis by modulating sugar metabolism. Yao et al. found that the Arabidopsis sus1/sus4 double mutant accumulated sucrose under hypoxia, whereas SuSy1/4 cleaved sucrose to provide substrates for glycolysis, sustaining energy supply under low oxygen [32]. In anaerobic environments, plants shift their energy metabolism toward glycolysis. SUS cleaves sucrose to generate fructose and UDP-G, supplying substrates for glycolysis and maintaining cellular ATP production.

SUS also plays a significant role in the growth and development of reproductive organs. In tomato, Duan et al. observed that SlSUS3 primarily functions in the cleavage direction in flowers and fruits, supplying carbon for the development of floral organ development and seed formation [33]. Silencing *SlSUS3* resulted in markedly reduced levels of glucose, fructose, sucrose, and starch in flowers, highlighting its critical role in carbon metabolism. Additionally, *SlSUS3* silencing resulted in a decrease in both flower number and the proportion of multi-petal flowers.

#### 3.1.4. Transport Proteins

In addition to core enzymes such as INV, SPS, and SUS, sucrose metabolism relies on the coordination of various auxiliary enzymes and transport proteins. UDP-glucose pyrophosphorylase (UGPase) provides the precursor UDP-glucose for sucrose synthesis; phosphoglucomutase (PGM) maintains the balance of phosphosugar pool; and pyrophosphatase (PPase) drives sucrose synthesis by hydrolyzing pyrophosphate. Meanwhile, sugar transport proteins mediate transmembrane sugar transport and compartmentalization. The primary functions of sugar transporters include facilitating sucrose loading and unloading in the phloem, as well as regulating the transport, distribution, and storage of soluble sugars between source and sink tissues [34,35].

### 3.2. Key Enzymes and Functions in Hexose Metabolism

Sucrose, the primary end product of plant photosynthesis, is transported to various tissues and organs. Upon import into the fruit, approximately half of the sucrose is hydrolyzed into fructose and glucose. These hexoses serve as essential carbon skeletons and energy sources for fruit growth and development. Prior to entering glycolysis, fructose and glucose are phosphorylated by fructokinase (FRK) and hexokinase (HXK), respectively, yielding fructose-6-phosphate (F6P) and glucose-6-phosphate (G6P). It is noteworthy that fructose can be phosphorylated by either HXK or FRK, whereas glucose is specifically phosphorylated by HXK [36,37].

#### 3.2.1. Fructokinase (FRK)

Fructokinase (FRK) is a key enzyme in fructose metabolism, catalyzing the phosphorylation of fructose to fructose-6-phosphate (F6P). This reaction directs carbon flow toward pathways such as glycolysis and starch biosynthesis [38].

A study by Zheng Bin et al. from our group investigated *MiFRK1* and *MiFRK2* in high and low-sugar mango varieties [39]. The correlation between *MiFRK1* and fructose content was cultivar-dependent, showing a negative correlation in ‘Renong No.1’ but a significant positive correlation in ‘Tainong No.1’. Although *MiFRK2* did not show a significant correlation, it was still involved in the regulation of fructose metabolism. These findings collectively demonstrate that both genes contribute to fructose content differences among mango varieties.

Yang et al. conducted in vitro enzymatic assays in apple and found that *MdFRK2* had significantly higher affinity for fructose than *MdFRK1* [40]. Overexpression of *MdFRK2* in apple leaves led to a 50–60% reduction in fructose concentration, accompanied by decreased glucose and sucrose levels, indicating enhanced fructose phosphorylation and reduced accumulation of free fructose. Similarly, Zhang et al. reported that knockout of *OsFRK3* in rice resulted in a 15–20% increase in sucrose and fructose concentrations in seeds [41]. Concurrently, starch content decreased significantly by 3.42–4.80%, suggesting that FRK facilitates starch synthesis by phosphorylating fructose and directing carbon toward this pathway. Disruption of *OsFRK3* consequently altered carbon partitioning within the seeds.

Furthermore, changes in FRK activity influence interconnected metabolic pathways including sucrose and sorbitol metabolism, forming a coordinated regulatory network. In transgenic apple overexpressing *MdFRK2*, key sorbitol metabolism genes *A6PR* and SDH1/2 were activated, promoting the conversion of sorbitol to fructose [40].

Concurrently, the activities of sucrose synthase and neutral invertase were suppressed, reducing the hydrolysis of sucrose to fructose and glucose. Ultimately, these metabolic shifts led to a 7–18% decrease in sucrose concentration in the leaves.

#### 3.2.2. Hexokinase (HXK)

HXK is a bifunctional enzyme in plants, serving both metabolic and regulatory roles. Metabolically, it catalyzes the phosphorylation of hexoses, initiating key pathways such as glycolysis, the pentose phosphate pathway, and starch biosynthesis through the conversion of glucose to glucose-6-phosphate (G6P). Additionally, it acts as a sugar sensor and signaling modulator, playing a central role in sugar metabolism, plant growth and development, and stress responses [42].

The regulation of sugar metabolism by HXK exhibits spatiotemporal specificity. In grape berries, HXK activity is high during early developmental stages when glucose/fructose levels are low but declines at ripening stage, coinciding with a significant increase in sugar content [31]. Liu et al. investigated HXK genes in sugarcane and observed differential expression between source and sink tissues [43]. Six genes, including *SsHXK1* and *SsHXK2*, were expressed at significantly higher levels in stems than in leaves, and their expression decreased gradiently as sugar accumulated in the stems, suggesting a role in sucrose transport and storage. The *SsHXK* family also displayed diverse expression patterns under drought and cold stress. After 6 h of cold treatment, 14 genes, including *SsHXK15* and *SsHXK16*, were significantly upregulated. Under drought conditions, 7 genes showed high expression after 10 days of drought, with 3 genes upregulated during recovery, supporting the involvement of HXK in stress adaptation. Similarly, in strawberry, the expression of *FpHXK1* increased significantly under polyethylene glycol (PEG)-induced drought stress [44]. Furthermore, inhibition of its kinase activity enhanced plant sensitivity to drought, confirming that HXK contributes to drought stress tolerance through both metabolic and signaling pathways.

### 3.3. Other Sugar Metabolic Pathways

Sorbitol metabolism represents a significant sugar alcohol pathway in plants, comprising primarily synthesis and degradation processes. In the synthesis pathway, glucose-6-phosphate (G6P) is reduced by NADPH under the catalysis of sorbitol-6-phosphate dehydrogenase (S6PDH) to yield sorbitol-6-phosphate (S6P), which is subsequently dephosphorylated by a phosphatase to form sorbitol. In the degradation pathway, sorbitol is oxidized by sorbitol dehydrogenase (SDH) to fructose, which subsequently enters glycolysis or other sugar metabolic pathways. Sorbitol serves not only as a major photosynthetic product and a form of carbon transport in Rosaceae plants but also participates in physiological processes, including osmotic regulation, antioxidant defense, and fruit development. The balance of sorbitol metabolism is therefore critical for plant growth, development, and environmental adaptation [45,46,47,48].

## 4. Regulatory Mechanisms of Soluble Sugar Metabolic Enzymes

The activity and expression of sugar metabolic enzymes in fruits are regulated through a multi-layered network, encompassing transcription control, epigenetic modifications, and signaling pathways. This network integrates endogenous hormones, environmental signals, and feedback from sugar metabolites to precisely govern sugar accumulation.

### 4.1. Transcriptional Regulation

Transcription factors (TFs) serve as central regulators by directly binding to the promoters of sugar metabolic enzyme genes or interacting with other regulatory proteins. Families such as myeloblastosis transcription factor (MYB), WRKY transcription factors (WRKY), NAM-ATAF1/2-CUC2 transcription factors (NAC), and ethylene-responsive factor (ERF) have been demonstrated to fine-tune the expression and activity of sugar metabolic enzymes.

#### 4.1.1. MYB

MYB transcription factors play dual regulatory roles, capable of either activating or repressing sugar metabolism-related genes. A summary of key MYB TFs and their functions is provided in Table 1.

In the activating mode, MYB TFs often form complexes with other transcription factors to enhance gene expression and accelerate sugar accumulation. Some are also induced by specific signals. For instance, Gao et al. found that in pear fruit, PuMYB12 acts as a transcriptional activator that directly binds to the promoter of the sucrose transporter gene *PuSUT4-like*, promoting its expression and enhancing sucrose accumulation [49]. In apple, Zhang et al. demonstrated that MdMYB305 directly binds to the promoters of sugar-related genes *MdCWI1*, *MdTMT2*, and *MdVGT3*, enhancing their transcriptional activity, accelerating sucrose hydrolysis, and thereby promoting sugar accumulation [50]. This activity is potentiated when MdMYB305 forms a complex with MdbHLH33, which stabilizes its binding to target promoters. In grape, VvMYB15 is induced by abscisic acid (ABA) and promotes the accumulation of glucose and fructose by directly binding to the promoter of the sugar transporter gene *VvSWEET15* [51].

In the repressing mode, MYB TFs can directly suppress target genes or function within repressive complexes. In melon, CmMYB44 negatively regulates starch synthesis by repressing the AGPase gene *CmAPS2-2,* thereby indirectly limiting sucrose accumulation [52]. Wei et al. reported that in strawberry, FaMYB44.2 functions as a transcriptional repressor by forming a complex with FabHLH3 and FaTTG1 to inhibit the transcriptional activity of the sucrose phosphate synthase gene *FaSPS3*, thereby reducing sucrose accumulation [53]. In contrast, FaMYB10 competitively binds to FabHLH3, alleviating the repression by FaMYB44.2 and activating the expression of sucrose synthesis-related genes. Zhang et al. also observed that when MdMYB10 binds to MdbHLH33, it attenuates the synergistic effect of MdbHLH33 on MdMYB305, leading to suppressed expression of sugar metabolic genes and decreased sugar content [50].

**Table 1:** Transcription factors MYB and their functions in sugar metabolism.

Species	Name	Regulatory Way	Reference
*Fragaria × ananassa*	*FaMYB44.2*	Negatively regulate sugar accumulation and inhibit the expression of the sucrose accumulation-related structural gene *FaSPS3.*	[53]
	*FaMYB10*	Inhibit FaMYB44.2 to promote sucrose accumulation.	[53]
*Malus domestica*	*MdMYB305*	Directly activate the expression of sugar-related genes (*MdCWI1*, *MdTMT2*, *MdVGT3*, etc.) to promote sugar accumulation.	[50]
	*MdMYB10*	When binding to MdbHLH33, it will weaken the synergistic effect of MdbHLH33 on MdMYB305, resulting in the inhibition of the expression of sugar metabolism genes and a decrease in sugar content.	[50]
	*MdMYB108*	Activate the expression of *MdPFK* to promote the accumulation of soluble sugars in the fruit pulp.	[54]
*Cucumis melo*	*CmMYB113*	When induced by ethylene, it can activate the expression of *CmSPS1* to regulate sucrose accumulation.	[25]
	*CmMYB44*	Inhibit the expression of *CmAPS2-2*, affecting starch accumulation and fruit flavor quality.	[52]
*Pyrus ussuriensis*	*PuMYB12*	Activate the expression of *PuSUT4-like* to promote sucrose accumulation.	[49]
*Vitis vinifera*	*VvMYB15*	Induced by ABA, VvMYB15 promotes glucose and fructose accumulation by directly binding to the *VvSWEET15* promoter.	[51]
	*VvMYB1*	Activate the expression of *VvTOR* to promote glucose accumulation.	[55]
*Citrus reticulata*	*CsMYB1*	When induced by drought signals, it activates the expression of *CwINV6*, promotes the activity of cell-wall invertase, accelerates the hydrolysis of sucrose, and increases the soluble sugar content.	[56]
*Solanum lycopersicum*	*SlMYB1R1*	SlMYB1R1, as a transcriptional activator, mediates the unloading and accumulation of sucrose in tomato fruits by directly regulating the expression of *SlSWEET12c*.	[57]
*Citrus sinensis*	*CsMYBS3*	*CsMYBS3* interacts with CsbHLH122 to form a transcription factor complex, which activates the expression of *CsSUT2* to accelerate sucrose accumulation.	[58]

#### 4.1.2. NAC

NAC transcription factors primarily function as positive regulators in sugar metabolism, often directly by activating related genes to promote sugar transport and accumulation (Table 2). In apple, MdNAC5 significantly enhances fructose accumulation through direct activation of the fructose transporter gene *MdTST2* and the neutral invertase gene *MdNINV6*, while also modulating the MdEIN3.4–MdSWEET15a regulatory module [20]. In grape, the expression of *VvNAC72* increases as the onset of fruit ripening, where it activates VvSWEET15 expression, thereby enhancing hexose transport capacity and promoting hexose accumulation in the fruit [59].

**Figure 4:**
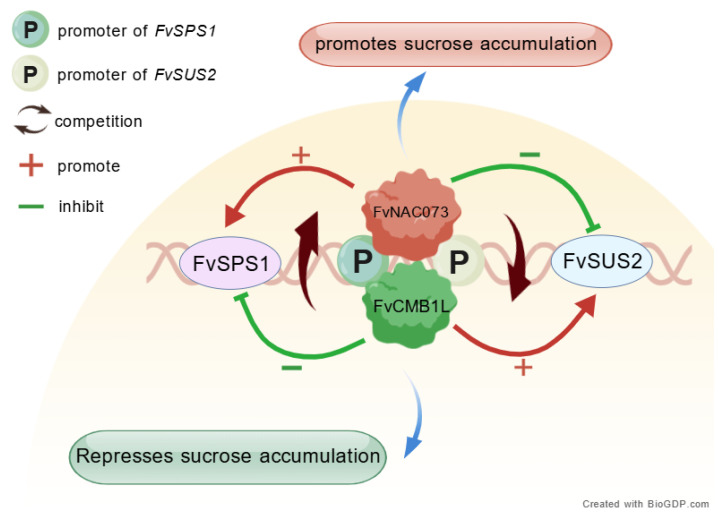
A proposed model for the antagonistic regulation of sucrose accumulation by FvNAC073 and FvCMB1L in strawberry. (This figure was created with BioGDP.com.) This schematic illustrates the competitive binding of transcription factors FvNAC073 and FvCMB1L to a shared promoter region of their target genes, leading to opposing effects on sucrose accumulation. The positive regulator FvNAC073, shown in green, enhances the expression of the sucrose synthesis gene *FvSPS1* while suppressing the sucrose cleavage gene *FvSUS2*, thereby promoting sucrose accumulation. Conversely, the repressor FvCMB1L, shown in red, competes with FvNAC073 for promoter binding. This competition inhibits the positive regulatory function of FvNAC073 and ultimately represses sucrose accumulation. The dynamic equilibrium between these two antagonistic transcription factors finely calibrates the sucrose level in fruit. Note: *FvSPS1*, Sucrose Phosphate Synthase 1; *FvSUS2*, Sucrose Synthase 2. P, promoter.

A notable finding by Xiao et al. first revealed an antagonistic relationship between NAC and MADS-box proteins in strawberry sucrose metabolism [60]. Specifically, FvNAC073 binds to the promoters of sucrose synthase genes *FvSPS1* and *FvSUS2*, promoting *FvSPS1* expression while repressing *FvSUS2*, thereby positively regulating sucrose accumulation. In contrast, the MADS-box protein FvCMB1L exerts an opposite effect by inhibiting sucrose accumulation. The two transcription factors maintain a dynamic balance through competitive binding to the same promoter region. This antagonistic regulatory module is schematically summarized in Figure 4.

**Table 2:** Transcription factors NAC and their functions in sugar metabolism.

Species	Name	Regulatory Way	Reference
*Malus domestica*	*MdNAC5*	Activating *MdTST2* and *MdNINV6* while regulating the MdEIN3.4-MdSWEET15a module to promote fructose accumulation.	[20]
*Vitis vinifera*	*VvNAC72*	Activating the expression of *VvSWEET15* promotes hexose accumulation.	[59]
*Fragaria × ananassa*	*FvNAC073*	FvNAC073 and FvCMB1L interact and competitively bind to the promoters of *FvSPS1* and *FvSUS2*, thereby antagonistically regulating sucrose accumulation.	[60]
*Musa nana*	*MaNAC19*	Activating the expression of *MaSPS1* promotes sucrose synthesis.	[61]
*Eriobotrya japonica*	*EjNAC25*	Activating the expression of *EjNI* accelerates the decomposition of sucrose into glucose and fructose.	[62]
*Citrullus lanatus*	*ClNAC68*	Inhibiting the expression of *ClINV* promotes sucrose accumulation	[63]
*Prunus persica*	*PpNAC1/5*	Activating *PpTST1/2* promotes sugar accumulation.	[64]

#### 4.1.3. WRKY

WRKY transcription factors play an important role in regulating sugar metabolism by integrating sugar and hormone signaling pathways, as summarized in Table 3. Huang et al. demonstrated that the grape VvWRKY22 is induced by fructose and ABA, but repressed by sucrose [65]. Overexpression of *VvWRKY22* led to reduced sucrose, glucose, and fructose levels, and its interaction with SnRK1 kinases (VvSnRK1.1/VvSnRK1.2) further modulated the expression of downstream sugar-metabolism-related genes.

In apple, Zhang et al. reported that MdWRKY126 significantly increased sucrose content by upregulating SPS activity and gene expression, while suppressing hexose transporter expression [66]. In postharvest apple fruit, Li et al. found that ethylene induces the expression of *MdWRKY32*, which in turn activates the transcription of *MdBam5* [67]. The encoded β-amylase catalyzes starch hydrolysis into maltose, which is further converted into glucose and fructose, thereby promoting soluble sugar accumulation.

**Table 3:** Transcription factors WRKY and their functions in sugar metabolism.

Species	Name	Regulatory Way	Reference
*Vitis vinifera*	*VvWRKY22*	Negatively regulates sugar accumulation.	[65]
*Malus domestica*	*MdWRKY126*	MdWRKY126 positively regulates sugar accumulation by upregulating the activity of SPS and the expression of related genes, while downregulating the activity of enzymes involved in sucrose decomposition and the expression of related genes.	[66]
	*MdWRKY32*	Activates the expression of *MdBam5* to promote sugar accumulation.	[67]
*Pyrus ussuriensis*	*PuWRKY31*	Activates the expression of *PuSWEET15* to promote sucrose accumulation.	[68]

#### 4.1.4. Other Transcription Factors

In addition to core TF families such as MYB and NAC, other transcription factors including ERF and basic helix-loop-helix (bHLH) are also involved in the regulation of sugar metabolism, as detailed in Table 4.

The ERF family plays dual regulatory roles in sugar metabolism. For example, in kiwifruit, AcERF182 activates the β-amylase gene *AcBAM3.5*, promoting starch degradation and soluble sugar accumulation [69]. CitERF16 enhances sucrose accumulation in citrus fruit by activating the expression of *CitSWEET11d* [70]. Conversely, VvERF105 acts as a repressor that suppresses *VvSWEET15* expression prior to grape ripening, thereby reducing hexose accumulation [59].

bHLH transcription factors regulate sugar homeostasis primarily through forming complexes with other TFs, although they can also independently modulate target genes. Zhai et al. observed that during the late maturation of sweet orange, *CsbHLH122* expression increases rapidly and acts synergistically with CsMYBS3 to activate *CsSUT2*, thereby driving rapid sucrose accumulation [58]. In apple, MdbHLH3 binds directly to the E-box element in the promoter of the phosphofructokinase gene *MdPFPβ*, activating its expression to promote the synthesis of fructose-6-phosphate (F6P) and sucrose, while concurrently enhancing the activities of sucrose synthase and fructokinase [71]. Similarly, in plum, PsbHLH58 activates the expression of *PsSUS4*, facilitating sucrose biosynthesis [72].

**Table 4:** Other transcription factors and their functions in sugar metabolism.

Transcription Factor	Species	Name	Regulatory Way	Reference
ERF	*Actinidia chinensis*	*AcERF182*	Regulates the starch degradation gene *AcBAM3.5* to promote the accumulation of sucrose and fructose.	[69]
	*Citrus reticulata*	*CitERF16*	Activates the expression of the sucrose transporter gene *CitSWEET11d* to promote sucrose accumulation.	[70]
	*Vitis vinifera*	*VvERF105*	Inhibits the expression of *VvSWEET15* to reduce hexose accumulation.	[59]
MADS-box	*Musa nana*	*MaMADS36*	Activating the expression of *MaBAM9b* promotes starch degradation and sugar accumulation.	[73]
PRE	*Pyrus ussuriensis*	*PuPRE6*	Inhibits the expression of *PuSUT4-like* and *PuMYB12*, negatively regulating sucrose accumulation.	[49]
ARF	*Fragaria × ananassa*	*FveARF2*	Inhibits the expression of the sucrose transporter gene *FaSUT1*, blocking sucrose transport.	[74]
CBF	*Malus domestica*	*MdCBF1/2*	Induced by low-temperature signals to activate the expression of the vacuolar sugar transporter *MdTST1/2*, promoting sugar accumulation.	[75]
ZAT	*Citrus reticulata*	*CitZAT5*	Activates *CitSUS5* to promote the decomposition of sucrose into glucose and fructose, and simultaneously activates *CitSWEET6* to promote fructose transport, increasing the proportion of hexose.	[76]
ABF	*Ziziphus jujuba*	*ZjABF1*	Induced by ABA signals to activate ZjSWEET11/18 for promoting sugar accumulation.	[77]
ANL	*Rosa roxburghii*	*RrANL2*	Activates the expression of the sucrose synthase gene *RrSUS3* to promote sugar accumulation.	[78]
bHLH	*Citrus sinensis*	*CsbHLH122*	CsMYBS3 interacts with *CsbHLH122* to form a transcription factor complex, activating the expression of *CsSUT2* and promoting sucrose accumulation.	[58]
	*Malus domestica*	*MdCIbHLH1*	*MdCIbHLH1* inhibits the expression of *MdFBP* and *MdPEPCK*, negatively regulating sugar accumulation.	[79]
		*MdbHLH33*	Forms a protein complex with MdMYB305 to promote the activation of MdMYB305 and regulate sugar accumulation.	[50]
		*MdbHLH3*	Activates the transcription of *MdPFPβ* to promote sucrose synthesis.	[71]
	*Prunus salicina*	*PsbHLH58*	Activates the expression of *PsSUS4* to promote sucrose synthesis; ethylene treatment upregulates the expression of *PsbHLH58*, thereby enhancing the activity of PsSUS4 and promoting sucrose accumulation.	[72]

### 4.2. Epigenetic Regulation

Epigenetic modifications provide a crucial layer of regulation for sugar metabolic enzymes by modulating chromatin accessibility or RNA stability, primarily through histone modification, non-coding RNAs, and DNA methylation.

Histone modifications influence gene transcriptional activity by modulating chromatin states. In pear fruit, the histone deacetylase PuHDAC9-like binds to the promoters of *PuMYB12* and *PuSUT4-like*, repressing their expression and reducing sucrose accumulation [49].

Non-coding RNAs orchestrate multiple aspects of fruit sugar metabolism—including sugar synthesis, transport, and partitioning—via complex regulatory networks. Long non-coding RNAs (lncRNAs) modulate target gene expression via cis- or trans-regulatory mechanisms, while microRNAs (miRNAs) mainly regulate sugar metabolism by targeting mRNA degradation or inhibiting translation efficiency. A study in apricot identified 36 lncRNAs and 34 miRNAs that potentially interact with sugar metabolism-related genes [80]. For instance, the *VIP1* gene in the MEbrown module appears to regulate sugar transporter expression through an lncRNA–mRNA network. A vacuolar proteomics study in citrus [81] further suggested that the expression of sugar transporter genes may be post-transcriptionally regulated by miRNAs. In sugarcane, miR172 and miR164 target AP2/ERF and NAC transcription factors, respectively, forming an ABA–miRNA–transcription factor cascade that regulates the expression of sucrose phosphate synthase and sucrose transporter genes [82].

Furthermore, allelic variation in apple SWEET (Sugars Will Eventually Be Exported Transporters) genes is associated with differential promoter methylation levels, suggesting that DNA methylation may affect their expression by modulating transcription factor binding [83].

In summary, histone modifications, non-coding RNAs, and DNA methylation collectively constitute a multi-layered epigenetic system for fine-tuning sugar metabolism in fruits. However, this field remains nascent, and future studies are needed to elucidate the specific molecular mechanisms and key regulatory elements involved.

### 4.3. Signaling Pathways

Hormone, environment, and sugar signals regulate the activity of transcription factors and epigenetic modifications, thereby forming a coordinated network that collectively controls the expression and activity of sugar metabolic enzymes.

#### 4.3.1. Hormonal Signaling

Phytohormones largely regulate sugar metabolism—including sucrose synthesis, transport, and starch degradation—primarily through transcription factor networks. Different hormones employ specific signaling pathways to influence the expression of sugar metabolism-related genes, forming synergistic or antagonistic regulatory circuits.

Methyl jasmonate (MeJA) regulates sugar metabolism by activating genes encoding sucrose synthesis-related enzymes. In tomato [84], MeJA acts as a signaling molecule that upregulates the activity and transcript levels of amylase, sucrose phosphate synthase (SPS), and sucrose synthase (SUS) (e.g., *SlSUS2*, *SlSUS3*, *SlSPS1*), thereby promoting starch degradation and sucrose synthesis. Concurrently, it downregulates acid and neutral invertases (AI and NI) activity, inhibiting sucrose cleavage into hexoses.

Abscisic acid (ABA) regulates sugar metabolism through phosphorylation cascades that modulate sugar transporter gene expression. In strawberry, ABA promotes the interaction between FaRIPK1 and FaTCP7, leading to the phosphorylation of the latter [85]. This phosphorylation relieves the transcriptional repression of sugar transporter genes *FaSTP13* and *FaSPT*, thereby enhancing sucrose and hexose transport and accumulation. In apple, ABA induces the transcription factor MdWRKY9 to bind the W-box element in the promoter of the sucrose transporter gene *MdSWEET9b*, activating its expression and promoting sucrose transport into fruits [86]. Furthermore, Li et al. demonstrated that root restriction in grapevines triggers an ABA-mediated VvGRIP55–VvMYB15–VvSWEET15 regulatory cascade [51]. VvGRIP55 activates *VvMYB15* transcription, which subsequently induces *VvSWEET15* expression, ultimately enhancing glucose and fructose accumulation. ABA also mediates “sugar–ABA” signaling crosstalk through transcription factors. In tomato and strawberry, ABA and sucrose synergistically induce ABA-stress-ripening (ASR) transcription factors [87]. These ASR proteins then activate sugar transporter genes and ABA biosynthetic genes, thereby promoting sugar accumulation and forming a positive “sugar–ABA” signaling feedback loop. Under drought stress in apple, ABA induces MdDREB2A to bind to the promoters of vacuolar sugar transporter genes *MdERDL6* and *MdTST* [75], facilitating hexose transport into vacuoles for accumulation while activating ABA biosynthetic genes *MdNCED1* and *MdNCED3* to enhance drought tolerance.

Ethylene, a key ripening hormone, regulates sugar metabolism through multiple transcription factors. In melon, ethylene induces CmMYB113 to activate the sucrose phosphate synthase gene *CmSPS1* and upregulate vacuolar proton pump activity to promote sucrose transport into vacuoles [25]. In tomato, the ASR protein, co-induced by ethylene and sucrose, promotes fruit ripening by activating hexose transporter gene expression [87]. In banana, ethylene induces MaMADS36, which activates genes involved in starch degradation and sucrose synthesis [73]. In postharvest apple, ethylene induces *MdWRKY32* expression as a ripening signal, stimulating starch hydrolysis [67].

#### 4.3.2. Environmental Signals

Environmental cues, including temperature and light, serve as key regulators of sugar metabolism in developing fruits by modulating the expression and activity of related metabolic enzymes.

Temperature exerts distinct effects on sugar metabolism. Under high-temperature stress, tomato plants significantly induces the expression and enzyme activity of the SPS gene [66], enhancing their thermotolerance. Conversely, low temperatures induce the expression of *MdCBF1/2* in apple. These transcription factors subsequently binds to the promoters of the sugar transporter genes *MdTST1/2*, promoting the accumulation of glucose and fructose [75].

Light signals regulate the expression of SWEET genes in tomatoes through transcription factors such as HY5, thereby influencing sugar transport [85]. Prolonged light exposure enhances sugar accumulation in apple flesh by activating MdMYB108 [54].

#### 4.3.3. Sugar Signal Feedback

Sugars such as sucrose and glucose not only serve as metabolic substrates but also function as signaling molecules that mediate feedback regulation by modulating kinase activity or transcription factor stability. In grapes, sucrose suppresses the expression of *VvWRKY22*, while fructose and ABA enhance its transcriptional activity, collectively forming a sugar-hormone regulatory network [65]. In plants, hexokinase (HXK), SNF1-related protein kinase 1 (SnRK1), and target of rapamycin (TOR) are key sugar-responsive signals involved in sugar metabolism [88]. Furthermore, in grapes, *VvTOR* is regulated by VvMYB1, and overexpression of *VvMYB1* activates this pathway and promotes glucose accumulation [55].

## 5. Summary and Outlook

Recent studies have revealed that the regulation of sugar metabolism in fruits is governed by a multi-layered regulatory network, rather than by a single factor. At the transcriptional level, transcription factors from families such as MYB, NAC, and WRKY modulate sugar metabolism-related genes by specifically binding to their promoters. At the epigenetic level, histone modifications, non-coding RNA regulation, and DNA methylation influence the expression of enzymatic genes at both pre- and post-transcriptional stages by altering chromatin states or mRNA stability. Meanwhile, hormonal, environmental, and sugar signals engage in intricate cascading interactions that intersect with transcriptional and epigenetic regulation, collectively governing sugar synthesis, transport, and accumulation. These mechanisms not only directly affect fruit sweetness and flavor but also contribute to overall fruit quality by modulating carbon allocation and cell turgor pressure.

In summary, while a fundamental regulatory framework for sugar metabolism regulation has been established, many aspects remain to be further explored. The rapid development of multi-omics technologies, such as metabolomics, proteomics, and single-cell sequencing, offers unprecedented opportunities to integrate multidimensional data, construct comprehensive regulatory networks, and identify novel regulatory nodes. In addition, current studies have focused on model fruits like apples and grapes, while the regulatory mechanisms underlying sugar metabolism in economically important subtropical specialty fruits like mangoes and lychees are relatively understudied. Future research could integrate the above multi-omics approaches to identify key regulatory factors unique to these species, elucidating their molecular functions and regulatory networks to provide new targets and theoretical support for quality improvement in specialty fruit trees.

At the molecular regulation level, current studies often concentrate on isolated aspects such as transcription, epigenetics, or signal transduction. Future work should employ advanced biotechnologies to clarify the patterns and sequence of multi-layered regulation, deepening our understanding of the cascade mechanism from “signal perception–transcriptional response–metabolic changes” for a more systematic comprehension of fruit quality formation.

From an applied perspective, the growing repository of regulatory modules and related metabolic enzyme genes provides a foundation for precision breeding. Gene editing technologies, particularly CRISPR/Cas9, could be employed to validate their effects on targeted regulation of sugar content and fruit quality, enabling targeted trait enhancement. Pursuing these research directions will not only deepen the theoretical understanding of fruit sugar metabolism but also accelerate the development of a high-quality, sustainable global fruit industry.

## Data Availability

Data sharing is not applicable to this article as no new data were created or analyzed in this study.
